# Distribution of PEN-FAST scores across a large health system: an opportunity for penicillin-allergy delabeling

**DOI:** 10.1017/ice.2026.10426

**Published:** 2026-05

**Authors:** Wesley J. Hoffmann, Shivani Patel, Shemual Tsai, Natalie A. Finch, Christy P. Su, Nicole A. Teran, Fadi Shehadeh, Muhammad Yasser Alsafadi

**Affiliations:** 1 Pharmacy, Texas Medical Center: Houston Methodist Hospital, Houston, USA; 2 Pharmacy, Houston Methodist West Hospital, USA; 3 Pharmacy, Houston Methodist Willowbrook Hospital, USA; 4 Medicine, Houston Methodist Hospital Physician Organization, USA

## Abstract

**Background:**

Penicillin allergy reporting is common in U.S. healthcare systems, but true allergies and clinically significant reactions are inaccurately reported. Validated tools like PEN-FAST score allow for structured risk assessment; however, many clinicians remain unfamiliar with how to utilize the score to inform decision-making and prescribing. Integrating the PEN-FAST tool into the electronic health record (EHR) admission workflow may promote awareness and improve clinical utility.

**Methods:**

We integrated the PEN-FAST tool into the admission navigator of our hospitals’ electronic health record to be completed by nursing staff. Over a seven-month period, completed PEN-FAST scores across our health system were analyzed to evaluate the overall opportunity for allergy assessment and delabeling. The study population consisted of patients with a documented penicillin class allergy and a completed PEN-FAST score. Patients with incomplete scores or responses marked as unknown for every item were excluded.

**Results:**

A total of 13,121 patients were included in the final evaluation. There were 10,309 (78.6%) patients with low-risk scores (PEN-FAST score of 0–2), indicating they were potential candidates for direct antibiotic challenges. The remaining 2,812 (21.4%) patients were categorized as high-risk (PEN-FAST score of 3+), who were likely not eligible for a challenge without prior skin testing.

**Conclusion:**

A substantial opportunity remains to improve the assessment and documentation of penicillin allergies throughout the healthcare system. Utilizing the electronic health record to prompt frontline staff to use validated risk assessment tools may improve documentation of allergies and support better management of patients with penicillin allergy labels.

## Summary

Our health system implemented the PEN-FAST score into EHR to be performed by nursing staff. We report the distribution of PEN-FAST scores among 13,121 patients and highlight opportunities available in healthcare systems to perform risk assessments and delabeling of penicillin allergies.

## Introduction

Penicillin allergy is the most documented drug allergy in U.S. electronic health records (EHR), with ∼ 10% of individuals reporting a history.^1^ However, most penicillin allergy labels are inaccurate.^2^ Access to formal allergy assessment is limited to a minority of healthcare systems. Most U.S. counties (81.5%) have no allergists available in local healthcare facilities, and the overall availability remains low overall (1.71 allergists per 100,000 people), constraining delabeling efforts.^3^ The PEN-FAST clinical decision rule has been validated and, in a randomized controlled trial, showed that it safely enables direct oral challenges of patients with scores <3, resulting in high delabeling rates.^4,5^ Additionally, the PEN-FAST score requires no special expertise or credentials to administer the questionnaire, nor specialized equipment as needed with penicillin skin testing, and can be administered by non-allergists, supporting scalable workflows.

Penicillin allergy labels can lead to significant prescribing consequences, often leading to the use of broader-spectrum therapies and increased reliance on non-beta-lactam antibiotics.^6^ Alternative agents are more likely to cause adverse effects as well as increase the risk of drug-resistant infections, such as Methicillin-resistant *Staphylococcus aureus* (MRSA), and *Clostridioides difficile*, which may lead to worse patient outcomes, including increased mortality, prolonged hospital stays, and higher health care costs. The economic burden resulting from these infections has a tremendous impact on institutional and societal costs.^7–9^

There is a significant opportunity in the inpatient setting for evaluation and management of penicillin allergies; however, the distribution of scores across a large patient population and precise volume of opportunity using a validated risk score has not yet been described. Houston Methodist implemented a modified PEN-FAST scoring tool within the EHR as part of the admission process that is performed by nursing staff. Nurses are the core component of frontline staff within hospitals and emergency departments, and this method of evaluating patients has been scaled up and implemented across our healthcare system at Houston Methodist, which encompasses over 2,700 inpatient beds and almost 150,000 inpatient admissions each year. We describe the distribution of PEN-FAST scores and identify opportunities to delabel penicillin allergy within a large, integrated health system.

## Methods

This is a retrospective, descriptive, cross-sectional analysis of PEN-FAST distribution among unique patients with a penicillin allergy label admitted to the Houston Methodist System between June 18, 2024, and February 1, 2025. The health system consists of an academic tertiary care medical center and six community hospitals, totaling more than 2,500 acute care beds. The PEN-FAST questionnaire was incorporated into the EHR admission navigator for use by nursing staff across the Houston Methodist System. We have previously reported a high concordance between nurse administered PEN-FAST scores and antimicrobial stewardship pharmacist verified classification, with a negative predictive value of a nurse-administered PEN-FAST score of 89.5%, which identifies patients at low-risk for true penicillin allergy.^10^ We pulled responses to questions from the assessment directly from internal Epic data using the Microsoft SQL Server Management Studio data sourcing tool and assigned the appropriate score as previously described by Trubiano and colleagues. One exception to the original score was individuals reporting severe non-IgE mediated reactions, such as Stevens-Johnson Syndrome (SJS)/Toxic Epidermal Necrolysis (TEN), Serum Sickness, Drug Rash with Eosinophilia and Systemic Symptoms (DRESS), and Acute Generalized Exanthematous Pustulosis (AGEP) were assigned a score of 4, consistent with the recommendation that patients who exhibit these reactions should not be challenged,^1^ thus providing a safety net scoring for these rare events. Answers reported as “unknown” reflect patient-reported data as documented by registered nurses and are not missing data (Supplemental Figure 1). Answers with a response of “unknown” were scored in this report according to the original score set forth by Trubiano and colleagues.^4^ The study period and methodology selected reflect the initial implementation and early sustainability of an EHR embedded allergy screening tool.

A Best-Practice Alert (BPA) was triggered in the EHR system for patients with a documented penicillin-class allergy label, prompting nurses to complete the penicillin assessment questionnaire (Supplemental Figure 2) within the admission navigator for patients admitted to the hospital, in observation status, the emergency department, or for same-day surgery. The final score was calculated by the EHR based on the responses. A second BPA was created to alert pharmacists to transcribe the score in the allergy section of the medical record after the nurse completed the PEN-FAST questionnaire (Supplemental Figure 3). This step functioned as a prompt to formally update the allergy record after nursing screening, due to constraints within the EHR and served as a safety and quality assurance. The score was then transcribed into the patient’s EHR within the “Allergies” section by the pharmacy staff, making the result accessible to all healthcare providers, and allowing allergy documentation to be incorporated into clinical decision making, such as allergy, and cross-reactivity alerts. Descriptions of PEN-FAST score interpretation was also included within the allergy comments field. Nursing and pharmacy staff education regarding the data surrounding penicillin allergy, the scoring tool, and its integration into Epic was provided as a required module with competency-based questions across the organization via the Houston Methodist learning management system. Provider education included training on locating PEN-FAST scoring documentation in Epic and application of the score to identify opportunities for delabeling via Pharmacy and Therapeutics newsletters.

**Figure 1.**
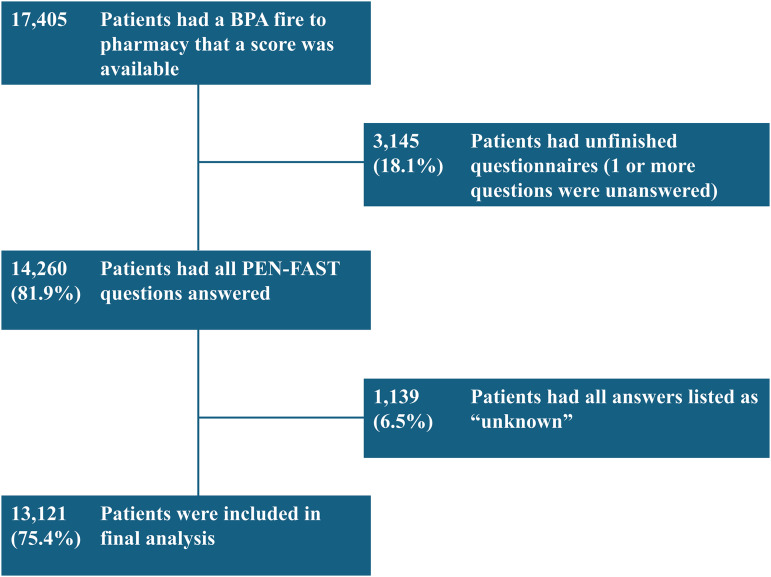
Flowchart for inclusion in analysis. Of 17,405 patients with a PEN-FAST score available, 3,145 were excluded for incomplete questionnaires and 1,139 for all responses marked “unknown,” leaving 13,121 patients in the final analysis. BPA, best practice alert.

Patients were included only if all components of the PEN-FAST assessment had been answered, and the score was available in the allergies field of the EHR, indicating a completed assessment. Any patient who had only partially responded to assessments (i.e., not all of the questions were answered) or whose score was not available in the allergy field was excluded from the analysis. Any patients who answered all scoring questions as “unknown” were excluded, as it was likely they couldn’t answer during acute hospitalization and the data would be unreliable. However, those who answered at least one question definitively, even if others were unknown, were included. The first eligible admission during the study period was used for patients with multiple encounters and multiple admissions. Details of the number of patients excluded and the rationale for exclusion are shown in Figure 1. The Houston Methodist Institutional Review Board reviewed the study protocol and granted this project exempt status as it is part of a quality assurance project.

## Results

From June 18, 2024, to February 1, 2025, nurses assessed 13,121 unique patients with a documented penicillin-class allergy using PEN-FAST score and pharmacists recorded this information in the allergy section of the EHR across the Houston Methodist Health System. A total of 3,145 (18.1%) patients had incomplete questionnaires, and 1,139 (6.5%) patients had all answers marked as “unknown” and were excluded (Figure 1). The demographics of these patients are shown in Table 1. Overall, 10,309 (78.6%) patients scored less than 3 and were considered low-risk for true penicillin allergy. The remaining 2,812 (21.4%) patients had a score that was 3 or higher and were considered high-risk (Table 2). The breakdown for each individual score is presented in Supplemental (Table 1). Responses to individual questions were as follows (Table 3):

**Table 1.** Baseline characteristics of the study population (N = 13,121)

Demographic	N = 13,121
Sex	
Female	9,103 (69.4%)
Male	4,018 (30.6%)
Age, median (IQR)	62.00 (44.00–74.00)
Race	
Asian	472 (3.6%)
Hispanic or Latino	1,998 (15.2%)
Non-Hispanic Black	2,578 (19.6%)
Non-Hispanic White	7,732 (58.9%)
Other / Unknown	341 (2.6%)
Underlying Medical Conditions	
Myocardial Infarction	1,864 (14.2%)
Congestive Heart Failure	2,962 (22.6%)
Peripheral Vascular Disease	3,904 (29.8%)
Cerebrovascular Disease	3,417 (26.0%)
Dementia	781 (6.0%)
Chronic Pulmonary Disease	5,216 (39.8%)
Rheumatic Disease	1,363 (10.4%)
Peptic Ulcer Disease	924 (7.0%)
Mild liver disease	2,723 (20.8%)
Diabetes without complications	4,333 (33.0%)
Diabetes with complications	2,554 (19.5%)
Hemiplegia or Paraplegia	680 (5.2%)
Renal disease	3,271 (24.9%)
Any malignancy	2,376 (18.1%)
Moderate or severe liver disease	293 (2.2%)
Metastatic Solid Tumor	1,554 (11.8%)
AIDS/HIV	98 (.7%)
Charlson score, median (IQR)	3.00 (1.00–7.00)
Site of Screening	
Emergency	4,562 (34.8%)
Outpatient Surgery	2,863 (21.8%)
Inpatient	3,735 (28.5%)
Observation	1961 (14.9%)

AIDS/HIV, acquired immunodeficiency syndrome/human immunodeficiency virus. IQR, interquartile range.

**Table 2.** Distribution of low risk vs high risk

Number of Unique Patients Assessed	N = 13,121
Low risk challenge (0-2)	10,309 (78.6%)
0	5,778 (44.0%)
1	3,325 (25.3%)
2	1,206 (9.2%)
High risk challenge (3+)	2,812 (21.4%)
3	2,074 (15.8%)
4+	738 (5.6%)

**Table 3.** Distribution of responses in individual questions in PEN-FAST questionnaire

Did reaction occur within the last 5 years?	N = 13,121
No	11,237 (85.6%)
Yes	1,536 (11.7%)
Unknown	348 (2.7%)
**Was the reaction anaphylaxis or angioedema?**	
No	8,819 (67.2%)
Yes	2,543 (19.4%)
Unknown	1,759 (13.4%)
**Did the reaction require treatment or hospitalization?**	
No	7,169 (54.6%)
Yes	3,328 (25.4%)
Unknown	2,624 (20.0%)
**Was the reaction ever reported as the following?**	
Rash, hives, or itching	9,174 (69.9%)
Unknown	1,957 (14.9%)
No	1,761 (13.4%)
Severe reaction, unspecified	153 (1.2%)
Serum sickness/drug induced fever	29 (.2%)
SJS/TEN	20 (.2%)
DRESS/AGEP	17 (.1%)
Interstitial nephritis/drug induced liver injury	10 (.1%)

SJS, stevens-johnson syndrome. TEN, toxic epidermal necrolysis. DRESS, drug reaction with eosinophilia and systemic symptoms. AGEP, acute generalized exanthematous pustulosis.

**Did the reaction occur in the past 5 years?**

Reactions within the previous 5 years were reported by 1,536 (11.7%) patients. In contrast, 11,237 (85.6%) patients reported that their reaction occurred more than 5 years ago.

**Was the reaction characterized by anaphylaxis or angioedema?**

Anaphylaxis or angioedema was reported by 2,543 (19.4%) patients, while 8,819 (67.2%) reported no signs or symptoms of either reaction.

**Did the reaction require treatment or hospitalization?**

A total of 3,328 (25.4%) patients stated they required treatment of some kind for their reaction, while 7,169 (54.6%) patients reported no need for treatment of their reaction.

**Was the penicillin allergy ever reported as one of the following? (Rash/Hives/Itching; Interstitial nephritis/Drug induced liver injury; serum sickness/drug induced fever; SJS/TEN; DRESS/AGEP; No; Unknown)**

Regarding rare but serious reactions, only 229 patients (1.8%) reported any of the following: SJS/TEN (20 patients), DRESS or AGEP (17 patients), serum sickness or drug-induced fever (29 patients), or interstitial nephritis or drug-induced liver injury (10 patients). Rash, hives, or itching were reported in 9,174 patients (69.9%). Of note, selections of “rash/hives/itching” or “unable to recall” were assigned a score of 0.

## Discussion

To our knowledge, this is the largest study to date evaluating the range of PEN-FAST scores, and the potential for penicillin allergy delabeling in an inpatient population. The incorporation of PEN-FAST evaluation to a broad patient population continues to be an implementation challenge for hospitals. Pharmacist-led manual evaluation of patients with penicillin allergies have been performed on small scales, confirming the utility of the PEN-FAST score in routine clinical practice.^11^ However, this study is the first to demonstrate the ability to scale the PEN-FAST evaluation across a health system and gain insight into PEN-FAST scores across a real-world patient sample. Penicillin allergies are prevalent across the healthcare continuum, and our data indicate that 3 in every 4 patients labeled with a penicillin allergy may be eligible for oral challenge and delabeling. Across a health system, this represents thousands of patients who could benefit from improved allergy documentation and antimicrobial stewardship.

Although penicillin allergies remain highly reported, the inpatient setting offers a unique opportunity to reassess these labels. The integration of the PEN-FAST assessment into the EHR enables frontline providers to identify low-risk patients during routine care. Our findings suggest that PEN-FAST can effectively support broader provider engagement in allergy assessment, beyond allergy and infectious disease specialists. The study demonstrates that real-world implementation of PEN-FAST can be accomplished when integrated into the EHR. Post-implementation monitoring of outcomes, such as adverse reactions following beta-lactam exposure and the impact on prescribing patterns are essential to ensure safety and detect unintended consequences. The tool should be positioned as an adjunct to clinical judgement, and its simplicity and design make it a practical addition to clinical workflows, encouraging timely and targeted interventions.

There are limitations to this study. First, it reflects the experience of a single health system, which, while geographically diverse within Houston, Texas, may not be representative of other regions or countries. Second, patient recall and response bias during hospitalization might influence the accuracy of PEN-FAST scoring, although prior work suggests the tool remains reliable in inpatient settings.^10^ Even with factors that may impact accuracy, the PEN-FAST assessment uses an objective methodology to collect clinically relevant data on reported allergies. The implementation of the assessment has allowed for the replacement of subjective allergy data with allergy-specific questions that support clinical decision-making and improved allergy documentation. Third, this analysis focuses on risk stratification rather than outcomes of oral challenges, unlike studies such as the PALACE trial, which may have been underpowered to detect severe reactions.^5^ Therefore, while PEN-FAST may help identify potential candidates for delabeling, we cannot confirm safety without direct challenge data.

Integrating the PEN-FAST tool into the EHR is highly feasible, particularly given its compatibility with existing workflows and its ease of use by nursing staff. Nurses, who comprise the majority of frontline healthcare workers, are well-positioned to administer the tool during patient admissions. Our data show that when completed by nursing staff, PEN-FAST maintains high accuracy, with a negative predictive value of 89.5% for ruling out high-risk scores. This supports its practical implementation without requiring extensive training or additional resources.

System-wide use of PEN-FAST has identified a large and accessible cohort of low-risk patients. An estimated three in four patients may be appropriate candidates for inpatient oral challenge. Identifying this low-risk set of patients provides a tangible opportunity to enhance antimicrobial stewardship and may improve patient outcomes through targeted delabeling efforts.

In summary, integrating PEN-FAST into routine EHR workflows represents a practical and scalable strategy to address inaccurate penicillin allergy labeling. This approach empowers a broader range of clinicians to participate in allergy reassessment, potentially improving antibiotic utilization, and reducing the burden of false penicillin allergy documentation across healthcare systems.

## Supporting information

Hoffmann et al. supplementary material Hoffmann et al. supplementary material

## Supplementary material

The supplementary material for this article can be found at https://doi.org/10.1017/ice.2026.10426.
