# Supplementary material for: Distribution of PEN-FAST scores across a large health system: an opportunity for penicillin-allergy delabeling
Source: Infect Control Hosp Epidemiol. 2026 Mar 23;47(5):494–8. doi: 10.1017/ice.2026.10426 (PMC13216811; doi:10.1017/ice.2026.10426)

Supplemental Online Content

Supplemental Table 1

| **PEN-FAST Score: 0** | **N = 5,778** |
| --- | --- |
| **Did reaction occur within the last 5 years?** |  |
| No | 5,778 (100%) |
| Yes | 0 (0%) |
| Unknown | 0 (0%) |
| **Was the reaction anaphylaxis or angioedema?** | |
| No | 5,569 (96.4%) |
| Yes | 0 (0%) |
| Unknown | 209 (3.6%) |
| **Did the reaction require treatment or hospitalization?** | |
| No | 5,778 (100%) |
| Yes | 0 (0%) |
| Unknown | 0 (0%) |
| **Was the reaction ever reported as the following?** | |
| Rash, hives, or itching | 4,265 (73.8%) |
| Unknown | 682 (11.8%) |
| No | 831 (14.4%) |
| Severe reaction, Unspecified | 0 (0%) |
| Serum sickness/drug induced fever | 0 (0%) |
| SJS/TEN | 0 (0%) |
| DRESS/AGEP | 0 (0%) |
| Interstitial nephritis/drug induced liver injury | 0 (0%) |
| **PEN-FAST Score: 1** | **N = 3,325** |
| **Did reaction occur within the last 5 years?** |  |
| No | 3,325 (100%) |
| Yes | 0 (0%) |
| Unknown | 0 (0%) |
| **Was the reaction anaphylaxis or angioedema?** | |
| No | 1,985 (59.7%) |
| Yes | 0 (0%) |
| Unknown | 1,340 (40.3%) |
| **Did the reaction require treatment or hospitalization?** | |
| No | 0 (0%) |
| Yes | 1,271 (38.2%) |
| Unknown | 2,054 (61.8%) |
| **Was the reaction ever reported as the following?** | |
| Rash, hives, or itching | 2,248 (67.6%) |
| Unknown | 914 (27.5%) |
| No | 163 (4.9%) |
| Severe reaction, Unspecified | 0 (0%) |
| Serum sickness/drug induced fever | 0 (0%) |
| SJS/TEN | 0 (0%) |
| DRESS/AGEP | 0 (0%) |
| Interstitial nephritis/drug induced liver injury | 0 (0%) |
| **PEN-FAST Score: 2** | **N = 1,206** |
| **Did reaction occur within the last 5 years?** |  |
| No | 397 (32.9%) |
| Yes | 658 (54.6%) |
| Unknown | 151 (12.5%) |
| **Was the reaction anaphylaxis or angioedema?** | |
| No | 736 (61.0%) |
| Yes | 397 (32.9%) |
| Unknown | 73 (6.1%) |
| **Did the reaction require treatment or hospitalization?** | |
| No | 1,206 (100%) |
| Yes | 0 (0%) |
| Unknown | 0 (0%) |
| **Was the reaction ever reported as the following?** | |
| Rash, hives, or itching | 843 (69.9%) |
| Unknown | 83 (6.9%) |
| No | 280 (23.2%) |
| Severe reaction, Unspecified | 0 (0%) |
| Serum sickness/drug induced fever | 0 (0%) |
| SJS/TEN | 0 (0%) |
| DRESS/AGEP | 0 (0%) |
| Interstitial nephritis/drug induced liver injury | 0 (0%) |
| **PEN-FAST Score: 3** | **N = 2,074** |
| **Did reaction occur within the last 5 years?** |  |
| No | 1,543 (74.4%) |
| Yes | 442 (21.3%) |
| Unknown | 89 (4.3%) |
| **Was the reaction anaphylaxis or angioedema?** | |
| No | 414 (20.0%) |
| Yes | 1,543 (74.4%) |
| Unknown | 117 (5.6%) |
| **Did the reaction require treatment or hospitalization?** | |
| No | 0 (0%) |
| Yes | 1,611 (77.7%) |
| Unknown | 463 (22.3%) |
| **Was the reaction ever reported as the following?** | |
| Rash, hives, or itching | 1,454 (70.1%) |
| Unknown | 227 (10.9%) |
| No | 393 (19.0%) |
| Severe reaction, Unspecified | 0 (0%) |
| Serum sickness/drug induced fever | 0 (0%) |
| SJS/TEN | 0 (0%) |
| DRESS/AGEP | 0 (0%) |
| Interstitial nephritis/drug induced liver injury | 0 (0%) |
| **PEN-FAST Score: 4+** | **N = 738** |
| **Did reaction occur within the last 5 years?** |  |
| No | 194 (26.2%) |
| Yes | 436 (59.1%) |
| Unknown | 108 (14.6%) |
| **Was the reaction anaphylaxis or angioedema?** | |
| No | 115 (15.6%) |
| Yes | 603 (81.7%) |
| Unknown | 20 (2.7%) |
| **Did the reaction require treatment or hospitalization?** | |
| No | 185 (25.1%) |
| Yes | 446 (60.4%) |
| Unknown | 107 (14.5%) |
| **Was the reaction ever reported as the following?** | |
| Rash, hives, or itching | 364 (49.3%) |
| Unknown | 51 (6.9%) |
| No | 94 (12.7%) |
| Severe reaction, Unspecified | 153 (20.7%) |
| Serum sickness/drug induced fever | 29 (3.9%) |
| SJS/TEN | 20 (2.7%) |
| DRESS/AGEP | 17 (2.3%) |
| Interstitial nephritis/drug induced liver injury | 10 (1.4%) |

SJS = Stevens-Johnson Syndrome; TEN = Toxic Epidermal Necrolysis; DRESS = Drug Reaction with Eosinophilia and Systemic Symptoms; AGEP = Acute Generalized Exanthematous Pustulosis

Supplemental Figure 1: PEN-FAST Risk Assessment Tool


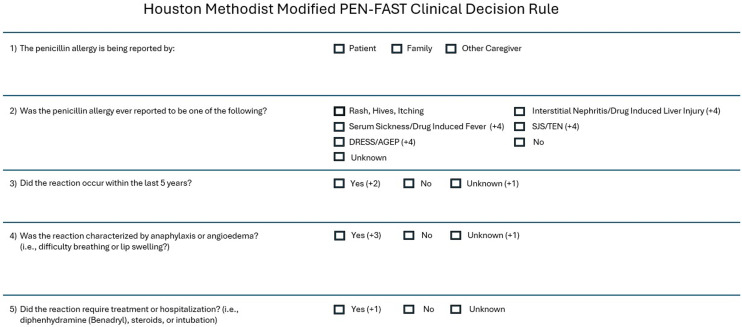


Supplemental Figure 2: Epic Penicillin Allergy Risk Assessment


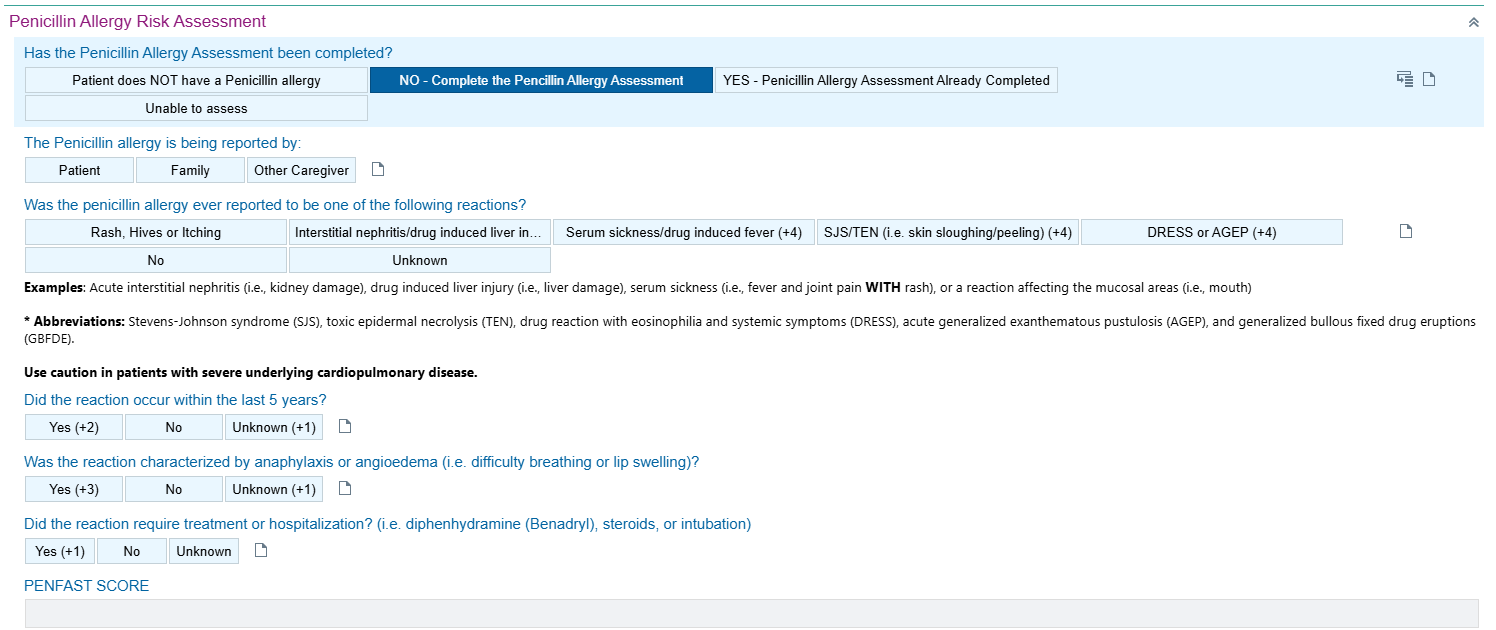


Scoring locally at Houston Methodist (shown above) is different than reported by Trubiano and colleagues, however all scores taken from our system were reverted back to original PEN-FAST scoring for this publication.

Supplemental Figure 3: Epic Penicillin Allergy Pharmacist BPA


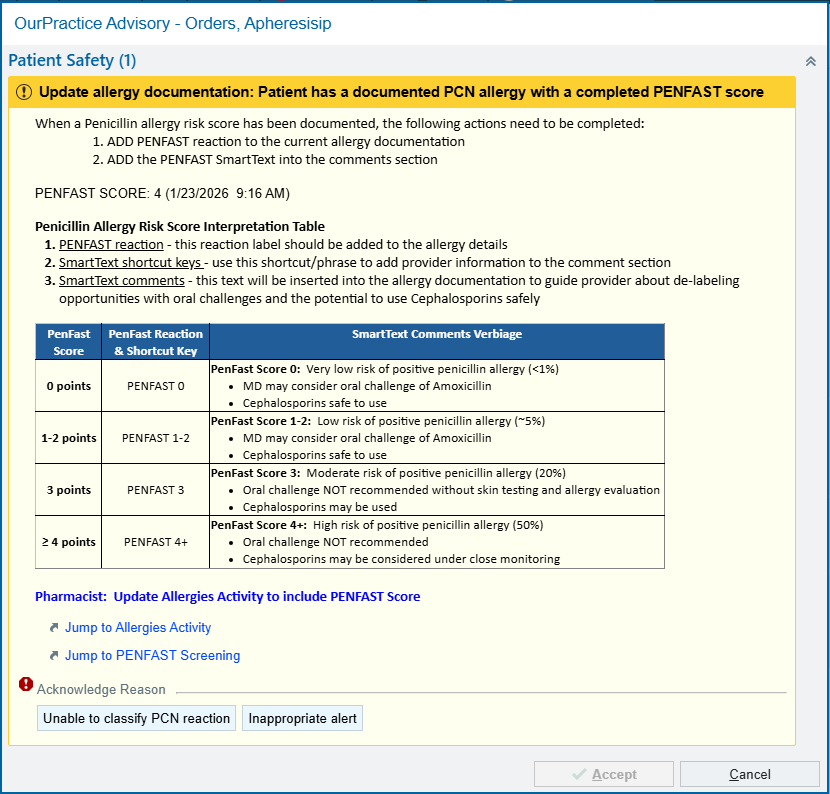

Supplement: Hoffmann et al. supplementary material [file S0899823X26104267sup001.docx]
